# Relationship between thyroid function and dietary inflammatory index in Hashimoto thyroiditis patients

**DOI:** 10.1097/MD.0000000000035951

**Published:** 2023-11-17

**Authors:** Si Chen, Yan Peng, Hao Zhang, Yandun Zou

**Affiliations:** a Department of Internal Medicine, Guangdong Women and Children Hospital, Guangzhou, China.

**Keywords:** diet, dietary inflammatory index, Hashimoto thyroiditis, inflammation, thyroid

## Abstract

Inflammation is closely related to the changes of thyroid function in Hashimoto thyroiditis patients. Certain nutrients or dietary habits can alter the levels of autoantibodies in Hashimoto thyroiditis. However, it remains unclear whether dietary inflammation affects thyroid function in patients with Hashimoto thyroiditis. The purpose of this study was to assess the relationship between dietary inflammation and thyroid function in Hashimoto thyroiditis patients using data from the National Health and Nutrition Examination Survey. We employed weighted multivariable linear regression, subgroup analyses, and interaction analysis to explore the relationship between thyroid function and dietary inflammatory index. We found that dietary inflammatory index was positively correlated with TSH and total T4. Interaction analysis found an interaction between urinary iodine concentration and FT3, but subgroup analysis for different levels of urinary iodine concentration did not get statistically significant results. Hashimoto thyroiditis patients with more pro-inflammatory diet habits had higher levels of TSH and TT4. In order to prevent hypothyroidism more effectively in patients with Hashimoto thyroiditis, it is essential to control dietary inflammation. However, it is still necessary to design a better prospective cohort study to verify the causal relationship.

## 1. Introduction

Hypothyroidism is a common endocrine disease, resulting in mild or severe clinical symptoms, which may be life-threatening. The most common cause of hypothyroidism is an autoimmune disease called Hashimoto’s thyroiditis (HT).^[1]^ The occurrence of HT is a result of environmental factors, genetic susceptibility, immune disorders and epigenetic factors. The histopathological features of HT include T and B cells infiltration and lymphoid follicle formation.^[2]^ Studies have shown that HT is associated with the development of other autoimmune diseases as well as papillary thyroid cancer. At present, the incidence of HT is increasing rapidly, especially among women of childbearing age, and may increase the risk of adverse pregnancy outcomes.^[3]^ Therefore, HT has become a major health problem of the whole society. Although we have conducted extensive studies on HT in the past, the exact pathogenesis and influencing factors are not fully understood.

Environmental factors such as pollution, iodine, sedentary lifestyles, stress, medications, unhealthy eating habits, and infection are triggers for many autoimmune diseases, as is HT.^[4]^ It has been confirmed that food is an important factor in the pathogenesis of Hashimoto Thyroiditis. High iodine intake, insufficient intake of selenium, iron, proteins, dietary fibers and unsaturated fatty acids may promote the development of HT.^[5]^ A large number of studies have shown that nutrition elements are closely related to Hashimoto thyroiditis. Benvenga et al^[6]^ found that iron and selenium are involved in the formation of T3 and T4, Zinc is important for T3 receptor activation and can influence thyroid function via other mechanisms. Studies have confirmed that insufficient intake of nutrients such as vitamins (A, B1, B5, B6, and C), proteins, and minerals (magnesium, sodium, potassium, phosphorus, and chromium) may also increase the incidence of HT.^[7]^

There is a large amount of evidence that proinflammatory food may cause intestinal imbalance, bacterial overgrowth, increase intestinal permeability and oxidative stress, and this inflammatory response will promote the occurrence of HT, so some scholars have proposed the concept of thyroid-gut axis.^[8]^ Kaličanin et al^[9]^ have demonstrated that the intake of animal fat and processed meat in patients with HT increase significantly, while the control group consume red meat, nonalcoholic beverages, whole grains and vegetable oil more frequently, and Triiodothyronine levels in patients with HT are positively correlated with the intake of vegetable oil. The dietary inflammation index was originally proposed by Shivappa et al^[10]^ in 2014 to assess the individual’s potential for dietary inflammation. Systemic inflammatory markers such as CRP, TNF-a, IL-6 and so on are positively correlated with dietary inflammatory index (DII) scores, and higher DII scores suggest stronger pro-inflammatory dietary habits.

However, current studies on diet and Hashimoto thyroiditis mostly focus on some specific nutrients or foods, and few studies on the relationship between overall dietary inflammation index and Hashimoto thyroiditis. At present, there are few studies on whether dietary inflammation index can affect thyroid function in Hashimoto patients. Therefore, this study aims to explore the relationship between dietary inflammation index and thyroid function in HT patients using data from the Public Database (NHANES).

## 2. Materials and Methods

### 2.1. Study population

**Figure 1.**
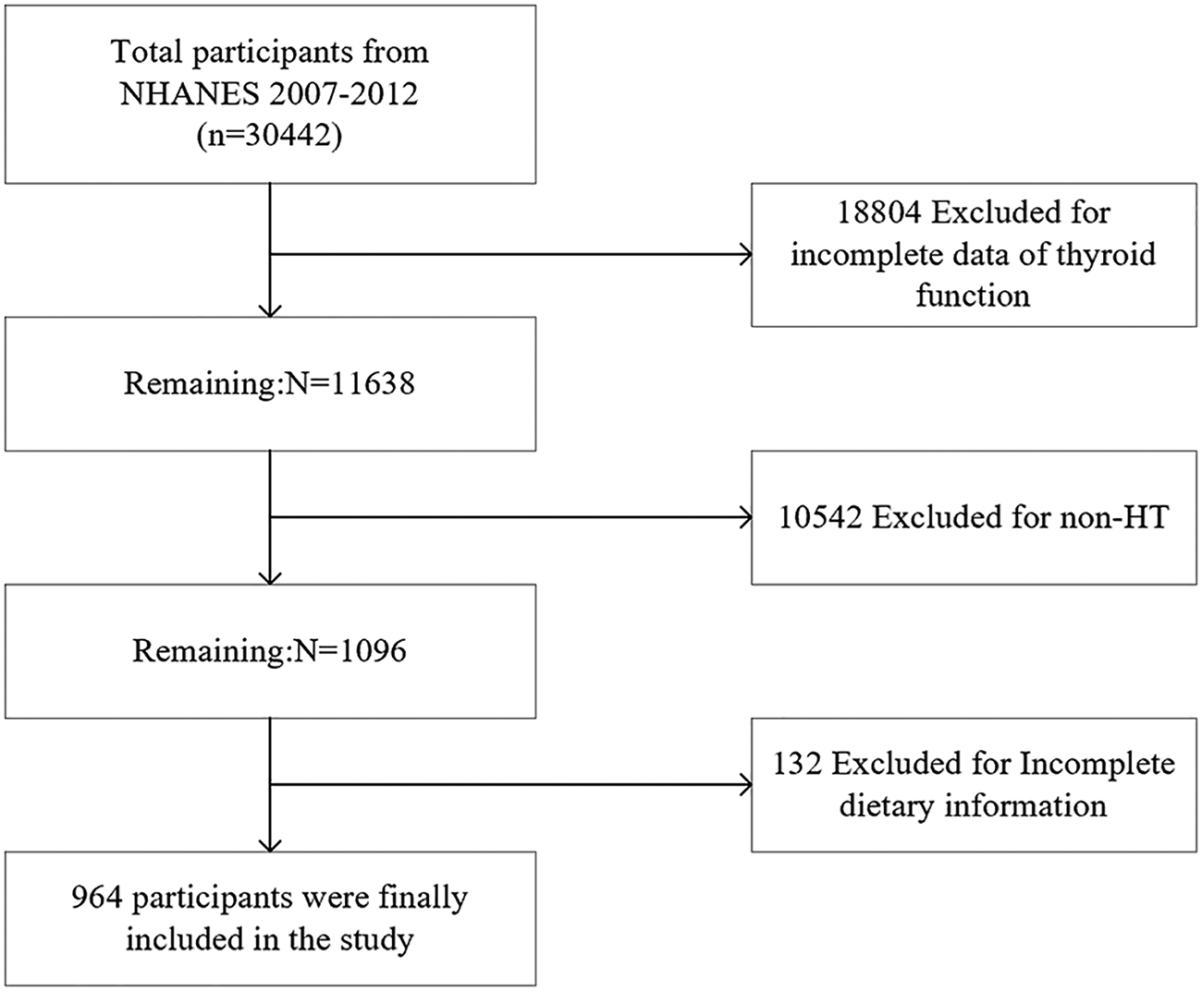
Flowchart of the study population.

We used data from the National Health and Nutrition Examination Survey (NHANES) database to conduct a cross-sectional study. Since only 3 cycles from 2007 to 2012 contained complete thyroid function data, we utilized dietary questionnaire data and thyroid laboratory testing data of these 3 cycles for analysis. The exclusion criteria were as follows: participants lacking partial thyroid data (n = 18,804); no-HT patients (n = 10,542), HT was defined as: TPOAb or TGAb positive (TPOAb > 9.0 IU/mL or TGAb > 115.0 IU/mL)^[11]^; participants lacking complete dietary data (n = 48); and participants lacking important covariates of alcohol consumption and hypertension data (n = 57). A total of 964 of 30,442 participants were included in the study in line with the inclusion criteria (Flowchart of the study population was shown in Fig. 1).

### 2.2. Calculation of DII

The DII score was calculated based on 24-hour dietary recall data. The specific calculation protocol was provided by Shivappa et al.^[10]^ Our calculation formula included 28 out of 45 food parameters, which were as follows: Energy, Protein, carbohydrates, fiber, fats, poly-unsaturated fatty acid, mono-unsaturated fatty acid, saturated fat, Cholesterol, Vitamin E, Vitamin A, beta-carotene, thiamin, riboflavin, Niacin, vitamin B6, folate, vitamin B12, Vitamin C, Vitamin D, magnesium, iron, zinc, Selenium, caffeine, Alcohol, n-3 fatty acid, n-6 fatty acid. According to previous experience, the DII value calculated using 28 food parameters is sufficient to predict the strength of an individual’s dietary inflammation.^[12]^

### 2.3. Assessment of thyroid function

The measurement of total T3 and T4 and free T3 and T4 were competitive binding immune-enzymatic assays. TSH was measured by a two-site, immune-enzymatic (“sandwich”) assay (third generation) and determined by a multi-point calibration curve (Lumi-Phos™ 530 was added). TPOAb and TgAb were measured by a sequential two-step immunoenzymatic “sandwich” assay, while Tg assay was a simultaneous one-step “sandwich” assay (Lumi-Phos™ 530 was added). Urine iodine concentration (UIC) was measured by ICP-DRC-MS (Inductively Coupled Plasma Dynamic Reaction Cell Mass Spectroscopy) to determine iodine conditions in participants.

### 2.4. Study covariates

We believed that the following variables may affect the statistical results, so we included them in the category of covariables: age (year), gender, race, education level, marital status, ratio of family income to poverty, UIC, body mass index (BMI, kg/m^2^), smoking status, hypertension, diabetes, alcohol consumption, and physical activities. The subjects included in the study include the following races: Mexican Americans, non-Hispanic whites, other Hispanics, non-Hispanic blacks, and other races. Educational level was divided into the following 5 categories: college graduate or above, some college, high school grade, 9–11th grade, and less than 9th grade. There were 6 types of marital status: married, widowed, divorced, separated, never married, living with partner. Alcohol consumption and smoking status were both classified as never, former and current.^[13]^ Physical activity was defined as yes (vigorous recreational activities or moderate recreational activities) and no (no moderate recreational activities).^[13]^ The classification of BMI was defined as follows: normal weight (<25), overweight (≥25, <30), and obese (≥30).^[14]^ Patients with the following conditions were defined as “hypertension”: systolic blood pressure ≥ 140 or/and diastolic blood pressure ≥ 90 mm Hg or having a history of hypertension.^[15]^ Participants with one of the following conditions were considered to have diabetes: a history of diabetes, use of insulin or oral hypoglycemic agents.^[15]^

### 2.5. Statistical analysis

In accordance with CDC guidelines, we performed all statistical analyses taking into account sample weights. Categorical variables were presented as percentages, while continuous variables were presented as mean ± standard deviation in this study. According to different levels of DII scores, the study subjects were divided into 3 groups. We employed the weighted student’s test (for continuous variables) and the weighted chi-square test (for categorical variables) to measure differences between 3 groups. Weighted multivariable linear regression was applied to investigate the association between DII and thyroid function (8 indexes were included: total and free T4 and T3, Tg, TgAb, TPOAb, and TSH). To further investigate the covariate influence on this association, we employed Model 1 (unadjusted), Model 2 (gender, age, race, education levels, marital status, and ratio of family income to poverty were adjusted), and Model 3 (fully adjusted model). To explore whether the conclusions of different populations are consistent, subgroup analyses were performed on variables, such as age, gender, BMI, UIC, hypertension, to evaluate the effect modification. Additionally, when performing subgroup analysis, all covariates except itself were adjusted. In addition, we tested the multicollinearity of the selected covariables by correlation analysis.

Since the proportion of missing data was small and not missing completely on random, NOMCAR option was used during the regression analysis. Statistical significance was set at *P* < .05. All statistical analyses were performed by the software package R (version 4.2.2, Lucent Technologies, Mount Jasmine, NJ).

## 3. Results

### 3.1. Baseline characteristics of participants

Sociodemographic and dietary characteristics and related covariates and dependent variables based on DII tertiles are presented in Table 1. After applying the selection and exclusion criteria, a total number of 964 people participated in the study. Male participants accounted for 32.4% of the total, while female participants accounted for 67.6%. The mean age was 51.43 years. DII scores ranged from −4.74 to 4.74, with an average value of 0.69. Among the 3 groups divided by DII levels, for laboratory examination, differences in TSH, and TgAb were statistically significant (*P* < .05). In terms of dietary information, there were significant differences in energy and protein intake among the 3 groups. The group with higher DII showed lower protein and energy intake. Regarding to sociodemographic information, covariables with statistical differences were as follows: gender, education level, ratio of family income to poverty, physical activity, smoking status, alcohol intake, BMI. People in DII tertile 3 group presented a higher percentage of lower level of education compared with other groups, and women seemed to be more likely to have a diet with a higher DII score. The DII tertile 3 group had a higher proportion of participants with lower ratio of family income to poverty and higher BMI. Participants in the highest DII group were more likely to be never drinkers or former drinkers, and current smokers. We also found that the higher the DII score, the lower the proportion of people engaged in physical activity.

**Table 1:** Weighted baseline characteristics of participants based on dietary inflammatory index.

	Total	DII Tertile 1	DII Tertile 2	DII Tertile 3	*P*
Age, mean ± SD (yr)	51.43 ± 16.21	51.21 ± 15.32	52.19 ± 16.53	50.81 ± 17.02	.702
Gender (%)					
Male	32.4	39.9	32.6	21.6	.001*
Female	67.6	60.1	67.4	78.4	
Race (%)					
Mexican American	7.4	6.8	7.9	7.4	.071
Other Hispanic	4.6	3.6	3.7	7	
Non-Hispanic White	78.8	82.4	80.8	71.4	
Non-Hispanic Black	4.3	2.3	4	7.5	
Other race	4.9	4.9	3.6	6.6	
Education level (%)					
Less than 9th grade	5.4	3.7	5.6	7.5	.012*
9–11th grade	9.8	7.8	8.8	13.8	
High school grad	24.2	21	21.9	31.3	
Some college	32.2	31.7	33	32	
College graduate or above	28.3	35.7	30.7	15	
Don’t know	0.1	0	0	0.4	
Marital status (%)					
Married	57.2	61.7	56.1	52.4	.249
Widowed	10.5	6.4	11.7	14.6	
Divorced	10.0	10.1	10.6	9.2	
Separated	1.6	1.4	2.3	1	
Never married	15.1	12.6	15	18.6	
Living with partner	5.7	7.8	4.4	4.3	
Ratio of family income to poverty, mean ± SD	3.2 ± 1.59	3.41 ± 1.65	3.39 ± 1.54	2.67 ± 1.44	<.001*
BMI (%)					
Normal	32.4	34.8	33.1	28.2	.011*
Overweight	34.7	42.2	30.6	29.1	
Obese	32.9	23	36.2	42.7	
Alcohol intake (%)					
Never	23.4	18.5	21.2	33	.009*
Former	10.2	8.8	9.1	13.5	
Current	66.4	72.7	69.8	53.5	
Smoking status (%)					
Never	55.7	63.1	44.9	58.6	.001*
Former	28.0	27.9	35.3	19.5	
Current	16.2	9.1	19.7	21.9	
Urinary iodine (%)					
Iodine deficient	34.1	36.3	28.7	37.6	.238
Normal	47.2	42.1	54.2	45.9	
Excessive iodine intake	18.7	21.7	17	16.5	
Physical activity (%)					
Yes	55.3	67	56.2	37.9	<.001*
No	44.7	33	43.8	62.1	
Hypertension status (%)					
Yes	32.9	30.5	35.9	32.4	.507
No	67.1	69.5	64.1	67.6	
Diabetes status (%)					
Yes	12.4	12	13.1	12.2	.918
No	87.6	88	86.9	87.8	
Energy, mean ± SD	1972.18 ± 859.94	2459.11 ± 935.35	1881.7 ± 644.00	1403.77 ± 532.59	<.001*
Protein intake, mean ± SD	77.39 ± 37.76	98.65 ± 40.87	72.62 ± 28.83	53.57 ± 24.20	<.001*
DII, mean ± SD	0.69 ± 1.96	−1.36 ± 1.11	1.15 ± 0.56	2.98 ± 0.62	<.001*
LBXATG, mean ± SD	75.30 ± 243.39	50.39 ± 180.12	97.80 ± 293.68	81.88 ± 248.79	.042*
LBXTPO, mean ± SD	180.32 ± 221.53	167.85 ± 190.71	184.40 ± 233.83	192.99 ± 245.46	.687
LBXT3F, mean ± SD	3.07 ± 0.47	3.10 ± 0.55	3.03 ± 0.39	3.07 ± 0.42	.29
LBDT4FSI, mean ± SD	10.37 ± 2.65	10.52 ± 2.36	10.28 ± 3.03	10.29 ± 2.52	.612
LBDTSH1S, mean ± SD	3.72 ± 7.95	2.60 ± 2.16	4.98 ± 11.55	3.77 ± 7.54	.003*
LBXTT3, mean ± SD	109.34 ± 27.03	111.33 ± 31.21	107.34 ± 23.55	109.00 ± 24.50	.312
LBXTT4, mean ± SD	7.92 ± 1.99	7.71 ± 1.51	7.99 ± 2.22	8.14 ± 2.23	.084
BMXBMI, mean ± SD	28.59 ± 6.71	27.37 ± 5.81	28.95 ± 6.98	29.84 ± 7.27	.007*

Mean ± SD for continuous variables: the *P* value was calculated by the weighted linear regression model. (%) for categorical variables: the *P* value was calculated by the weighted chi-square test.

DII = dietary inflammatory index.

*indicates *P* is less than 0.05.

### 3.2. The relationship between thyroid function and DII

We used weighted multivariable linear regression to explore the relationship between thyroid function and DII in Hashimoto thyroiditis patients (Table 2). Our study revealed that DII was positively correlated with total T4 and TSH (Model 1, β = 0.1237, 95% confidence interval [CI]: 0.0536, 0.1938, *P* = .0009; β = 0.2599, 95% CI: 0.0596, 0.4602, *P* = .0121); (Model 2, β = 0.1206, 95% CI: 0.0328, 0.2084, *P* = .0083; β = 0.238, 95% CI: 0.0492, 0.4268, *P* = .0147); (Model 3, β = 0.0947, 95% CI: 0.0017, 0.1876, *P* = .0462; β = 0.2154, 95% CI: 0.0043, 0.4265, *P* = .0457). Table 3 shows correlation between all variables included in the regression models.

**Table 2:** Associations between thyroid function and DII in Hashimoto thyroiditis patients.

DII	Free T3 (pg/mL)	Free T4 (pmol/L)	TSH (µ/L)	Total T3 (ng/dL)	Total T4 (µg/dL)
Model 1	−0.0023	−0.035	0.2599	−0.2546	0.1237
	−0.0199, 0.0154	−0.1416, 0.0716	0.0596, 0.4602	−1.5305, 1.0211	0.0536, 0.1938
	0.797	0.513	0.0121	0.69	0.0009
Model 2	0.0006	−0.0258	0.238	−0.4116	0.1206
	−0.01838, 0.0196	−0.1312, 0.0797	0.0492, 0.4268	−1.6645, 0.8414	0.0328, 0.2084
	0.95	0.6242	0.0147	0.511	0.0083
Model 3	−0.0064	−0.0407	0.2154	−1.4031	0.0947
	−0.0278, 0.0149	−0.1588, 0.0774	0.0043, 0.4265	−2.9393, 0.1331	0.0017, 0.1876
	0.5442	0.4889	0.0457*	0.0722	0.0462*

Model 1: unadjusted.

Model 2: gender, age, race, education levels, marital status, and ratio of family income to poverty were adjusted.

Model 3: fully adjusted model.

*indicates *P* is less than 0.05.

**Table 3:** Correlation between variables included in the regression models.

	1	2	3	4	5	6	7	8	9	10	11	12	13	14
Gender	–													
Age	−0.014	–												
Race	0.151	0.114	–											
Education	0.014	−0.114	0.239	–										
Marital status	0.016	−0.284	−0.067	−0.040	–									
FIR	−0.064	0.004	0.113	0.460	−0.194	–								
BMI	−0.037	0.009	−0.070	−0.027	−0.009	−0.054	–							
Urinary iodine	−0.112	0.103	−0.017	−0.070	−0.099	−0.021	0.105	–						
Alcohol intake	−0.245	−0.210	0.009	0.193	0.056	0.214	−0.063	0.010	–					
Diabetes status	−0.001	−0.202	−0.067	0.044	0.052	0.062	−0.211	−0.035	0.151	–				
Hypertension	0.086	−0.393	−0.087	0.044	0.105	0.002	−0.222	−0.063	0.090	0.300	–			
Smoking	−0.116	0.002	0.040	−0.099	0.075	−0.097	−0.023	−0.018	0.217	0.010	−0.005	–		
PA	0.045	0.203	−0.089	−0.263	0.001	−0.269	0.122	0.034	−0.168	−0.121	−0.10	0.101	–	
DII	0.129	0.114	−0.028	−0.179	0.042	−0.224	0.151	−0.020	−0.158	−0.087	−0.04	0.046	0.207	–

BMI = body mass index, DII = dietary inflammatory index, FIR = ratio of family income to poverty, PA = physical activities.

**Figure 2.**
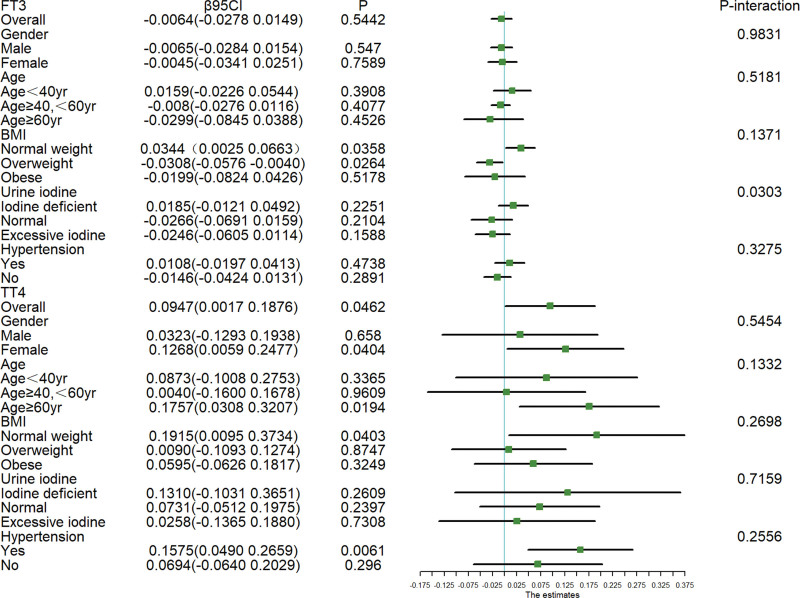
Stratified associations between thyroid function and dietary inflammatory index. Adjusted for gender, age, race, education level, marital status, ratio of family income to poverty, BMI, urinary iodine, alcohol intake, diabetes status, hypertension status, smoking, and physical activities. BMI = body mass index.

Subgroup and interaction analyses were performed using selected variables (age, gender, smoking status, BMI, history of diabetes, history of hypertension, and urinary iodine and physical activity) to further assess the association between thyroid function and DII (The results were shown in Fig. 2). Subgroup analyses of all the above variables did not significantly alter the relationship between TT3, FT4, TSH and DII, so we did not present these results in the table. None of these variables significantly altered the association between TT4 and DII: gender (*P* interaction = .5454), age (*P* interaction = .1332), BMI (*P* interaction = .2698), hypertension (*P* interaction = .2556), and urine iodine (*P* interaction = .7159).The following variables did not affect the relationship between FT3 and DII: gender (*P* interaction = .5454), age (*P* interaction = .5181), BMI (*P* interaction = .1371), and hypertension (*P* interaction = .3275). However, there was a significant interaction between serum FT3 levels and urinary iodine concentration (*P* = .0303). In other words, urinary iodine concentration influenced the relationship between FT3 and DII. In Iodine deficient group, FT3 was positively correlated with DII (β = 0.0185; 95% CI −0.0121, 0.049), while FT3 was negatively correlated with DII in normal and excessive iodine groups (β = −0.0266; 95% CI −0.0691, 0.0159) (β = −0.0246; 95% CI −0.0605, 0.0114). But neither of these correlations were statistically significant.

## 4. Discussion

Our study utilized a multiple linear regression model to assess the relationship between DII and thyroid function in Hashimoto thyroiditis patients. A total of 964 Hashimoto Thyroiditis patients were included in the study, and the positive correlation between TSH, TT4 and DII was statistically significant. After adjusting for all covariates, this positive correlation still existed. Subgroup analysis stratified by BMI showed that FT3 levels may significantly increase in normal weight individuals with higher DII scores or overweight individuals with lower DII scores. Subgroup analyses of TT4 and DII showed that the positive association between TT4 and DII was more significant in female, age ≥ 60 years, normal weight, and hypertensive patients. In all subgroup analyses, we calculated the interaction of variables, and we found that the following variables including gender, age, BMI and hypertension did not affect the relationship between DII and thyroid function. Only the P-interaction between urinary iodine and DII was less than 0.05, indicating that urinary iodine concentration affected the relationship between DII and FT3.

There were numerous studies on the relationship between certain dietary habits or nutritional elements and HT, but the results were controversial. Some studies showed that proper diet can improve thyroid function and reduce the reactivity of thyroid autoantibodies in HT.^[16]^ A study on iron and thyroid function in female population found that serum free T4 concentrations were significantly lower in the iron-deficient group than in the iron-deficient group, confirming that iron deficiency was associated with hypothyroidism.^[17]^ A study conducted by Krysiak et al^[18]^ indicated that a gluten free diet decreased serum TPO-Ab and Tg-Ab titers, but had no significant effect on thyrotropin and free thyroid hormones. According to a study directed by Roberta, increased adherence to the Mediterranean Diet was independently associated to a slightly reduced thyroid function, but still within the reference range for free T3 and T4 serum levels.^[19]^ A study on the relationship between smoking and HT suggested that smoking may increase the risk of hypothyroidism in Hashimoto’s patients.^[20]^ However, some studies showed that different diets have little effect on thyroid function. Metso et al^[21]^ found that there was no statistical difference in TSH and fT4 levels between the gluten-free group and the control group.

The results of the BMI-stratified subgroup analysis indicated that FT3 was positively correlated with dietary inflammation index in the normal weight group, while in the overweight group, this correlation was negative. Many previous studies had confirmed that weight is one of the factors affecting thyroid function. A cohort study confirmed that overweight and obesity might lead to higher free T3 levels.^[22]^ Kitahara et al^[23]^ found that BMI and waist circumference were positively associated with serum TSH levels and free T3 but not free T4 among euthyroid adults. A prospective study indicated that higher free T3 level was a consequence rather than a cause of weight gain.^[24]^ A study investigating whether obesity and dietary habits affect high oxidative stress in women with HT showed that women in the overweight or obese group, the low fruit intake group, and the low vegetable intake group had higher serum total lipid peroxidation levels.^[25]^ The mechanism by which overweight or obesity causing an increase in T3 is not fully understood, but it had been reported that leptins secreted by adipose cells may stimulate the synthesis and secretion of TSH and contribute to T4 to T3 conversion by deiodinases.^[26]^

The results of stratified subgroup analysis of urinary iodine showed that FT3 was positively correlated with DII when iodine was deficient, and negatively correlated with DII when iodine was sufficient or excessive. Numerous previous studies had confirmed that iodine played an irreplaceable role in the proper functioning of the thyroid gland. Iodine is important not only for thyroid hormone synthesis, but also for the induction and regulation of thyroid autoimmunity.^[27]^ The association between iodine intake and the presence of circulating thyroid antibodies is complex with iodine intake both below and above the recommended level being associated with an increase in circulating antibodies.^[16]^ The possible reasons for the increase of circulating antibodies caused by high iodine concentration were as follows: the strong immunogenicity of high iodinated Tg may trigger an immune response against the thyroid gland; excessive iodine intake increased the expression of intercellular adhesion molecule-1 on thyroid cells, leading to monocyte infiltration and accelerated inflammation.^[27,28]^ Therefore, it is best to ensure that iodine intake is within the recommended levels, and a quantifiable indicator is to maintain a median urinary iodine of 100 to 200 µg/L.^[29]^

According to the calculation scheme of dietary inflammation index provided in previous literature, food components were divided into pro-inflammatory and anti-inflammatory components. A total of 28 food parameters were covered in this study. After reviewing the literature, we found that there had been some studies exploring the relationship between some food parameters and Hashimoto thyroiditis or thyroidism, but there were still some food parameters that had no relevant studies at all. A study on the effects of diet reduction on thyroid function in Hashimoto thyroiditis patients confirmed that reduced energy intake can reduce TSH levels and increase FT3 and FT4 levels.^[30]^ Using animal models, the study by Shao et al^[31]^ has demonstrated that 24 weeks of high-fat diet decreased serum T4 and T3 levels in parallel with elevated concentrations of thyrotropin (TSH), indicating hypothyroidism. Animal studies indicated that vitamin A interfered with peripheral thyroid hormone metabolism. It was observed in the mouse model that vitamin A deficiency reduced the binding and uptake of T3 in tissues, reduced the transformation of T4 to T3 in the liver, and thus increased the thyroid hormones in the blood.^[32]^ A study on patients with autoimmune hypothyroidism showed that 46% of them were deficient in vitamin B12, and Vitamin B12 levels was negatively correlated with TPOAb antibodies.^[33]^ Numerous studies had been conducted on the role of vitamin D in HT, but the current conclusions were controversial. some studies found an association between vitamin D deficiency and the presence of thyroid autoantibodies. On the contrary, some studies showed no differences in vitamin D levels in patients with HT and the healthy subjects.^[34]^ A study involving 1257 Chinese participants confirmed that severely low serum magnesium levels were associated with an increased rate of TGAb positivity, HT, and hypothyroidism.^[35]^ Iron is necessary in the production of thyroid hormones, and its deficiency may result in a reduction in the synthesis of thyroid hormones and an increase in TSH level and thyroid volume.^[36]^ There’s an increased risk of hypothyroidism when the body is deficient in zinc because zinc is one of the trace elements necessary for the synthesis of thyroid hormone.^[37]^ A meta-analysis found that selenium supplementation reduced serum tpo antibodies levels in patients with chronic autoimmune thyroiditis treated with L-T4 after 3, 6, and 12 months.^[38]^ However, there were different conclusions. A RCT study exploring the effect of selenium supplementation on AITD in pregnant women found no difference in the magnitude of TPO reduction between the selenium supplementation group and the placebo group.^[39]^ There were no studies that specifically exploring the relationship between coffee intake and the onset of thyroid disease in humans. Studies in rat models had shown that caffeine consumption led to a decrease in TSH levels, which in turn led to a decrease of T3 and T4.^[40]^ Two studies from Denmark both found that moderate alcohol consumption reduced the risk of hypothyroidism in autoimmune thyroiditis.^[41,42]^

The main inflammatory factors involved in the calculation of dietary inflammation index included interleukin-6 (IL-6) and tumor necrosis factor-α. A study observing changes of inflammatory factors in autoimmune thyroid disease found that the hypothyroidism group had higher levels of serum IL-6 and tumor necrosis factor-α.^[43]^ A study by Papanas et al^[44]^ exploring the potential role of interleukin-6 in the treatment of Hashimoto’s disease by thyroid hormone replacement showed that serum IL-6 levels were positively correlated with thyroxine replacement dose. The above research conclusions were consistent with part of our results that individuals with a higher DII had higher levels of TSH. Paradoxically, our study found a positive correlation between DII and TT4. Another study examining the relationship between dietary inflammation and thyroid function in American adult men found that men who adhered to a pro-inflammatory diet had higher overall T4 levels, which was consistent with our findings.^[45]^ A possible explanation for the increase of TT4 levels due to dietary inflammation is described below. The higher the DII, the higher the concentration of cytokines such as IL-6 produced by the body. Studies had shown that IL-6 can inhibit the transformation of T4 to T3 mediated by deiodinase,^[46]^ resulting in higher levels of TT4 in peripheral blood. Of course, this is just a conjecture. More diet-based studies are needed to explore more convincing mechanisms of changes in thyroid function in HT patients.

The highlight of this paper is that we introduce an objective index of dietary inflammation to evaluate the level of inflammation caused by individual eating habits. Previous studies on thyroid function and DII were conducted in normal population. Most of studies on Hashimoto’s thyroid and DII were about the influence of dietary inflammation on the prevalence of HT. Few studies on the relationship between diet and thyroid mostly focused on a single food, a single nutrient element or a certain diet habit, with little research on the relationship between the quantitative indicators of individual diet-induced inflammation and thyroid. To our knowledge, this is the first study to explore the effect of DII on thyroid function in patients with Hashimoto thyroiditis. However, there are still some limitations to our study. This is a cross-sectional study, so we can’t make causal inferences. A large prospective Cohort study is needed to further confirm our results in the future. DII calculations based on 24-h dietary recall, annual family income, and martial and smoking statuses were obtained from questionnaires in NHANES; hence, recall bias and social desirability bias are inevitable. NHANES did not contain information on medications that could probably affect thyroid functions. The insufficiency of candidate basic and clinical studies made it very hard to clearly explain the association between DII and related hormone fluctuations.

## 5. Conclusions

Hashimoto thyroiditis patients with more pro-inflammatory diet habits had higher levels of TSH and TT4. In order to prevent hypothyroidism more effectively in patients with Hashimoto thyroiditis, it is essential to control dietary inflammation. However, it is still necessary to design a better Prospective cohort study to verify the causal relationship.

## Acknowledgments

We appreciate the colleagues and the participants of NHANES for their effort.

## Author contributions

**Conceptualization:** Yandun Zou.

**Data curation:** Si Chen, Yan Peng.

**Formal analysis:** Si Chen.

**Funding acquisition:** Si Chen.

**Investigation:** Si Chen, Yan Peng.

**Methodology:** Si Chen, Yan Peng.

**Project administration:** Si Chen, Hao Zhang, Yandun Zou.

**Resources:** Si Chen, Hao Zhang.

**Software:** Si Chen, Yan Peng, Hao Zhang.

**Supervision:** Si Chen, Yandun Zou.

**Validation:** Si Chen, Hao Zhang.

**Visualization:** Si Chen, Hao Zhang, Yandun Zou.

**Writing – original draft:** Si Chen.

**Writing – review & editing:** Si Chen, Yandun Zou.
